# Inosine Triphosphate Pyrophosphatase Dephosphorylates Ribavirin Triphosphate and Reduced Enzymatic Activity Potentiates Mutagenesis in Hepatitis C Virus

**DOI:** 10.1128/JVI.01087-18

**Published:** 2018-09-12

**Authors:** Kristina Nyström, Paulina H. Wanrooij, Jesper Waldenström, Ludmila Adamek, Sofia Brunet, Joanna Said, Staffan Nilsson, Megan Wind-Rotolo, Kristoffer Hellstrand, Helene Norder, Ka-Wei Tang, Martin Lagging

**Affiliations:** a Department of Infectious Diseases/Virology, Institute of Biomedicine, Sahlgrenska Academy, University of Gothenburg, Gothenburg, Sweden; b Department of Medical Biochemistry and Biophysics, Umeå University, Umeå, Sweden; c Department of Mathematical Sciences, Chalmers University of Technology, Gothenburg, Sweden; d Department of Pathology and Genetics, Institute of Biomedicine, Sahlgrenska Academy, University of Gothenburg, Gothenburg, Sweden; e Bristol-Myers Squibb, Princeton, New Jersey, USA; f Wallenberg Centre for Molecular and Translational Medicine, University of Gothenburg, Gothenburg, Sweden; University of Southern California

**Keywords:** hepatitis C virus, *ITPA*, ITP pyrophosphatase, ITPase, ribavirin, mutagenesis, inosine triphosphate pyrophosphatase, mutagenesis

## Abstract

A third of humans carry genetic variants of the ITP pyrophosphatase (ITPase) gene (*ITPA*) that lead to reduced enzyme activity. Reduced ITPase activity was earlier reported to protect against ribavirin-induced hemolytic anemia and to diminish relapse following ribavirin and interferon therapy for hepatitis C virus (HCV) genotype 2 or 3 infections. While several hypotheses have been put forward to explain the antiviral actions of ribavirin, details regarding the mechanisms of interaction between reduced ITPase activity and ribavirin remain unclear. The *in vitro* effect of reduced ITPase activity was assessed by means of transfection of hepatocytes (Huh7.5 cells) with a small interfering RNA (siRNA) directed against *ITPA* or a negative-control siRNA in the presence or absence of ribavirin in an HCV culture system. Low ribavirin concentrations strikingly depleted intracellular GTP levels in HCV-infected hepatocytes whereas higher ribavirin concentrations induced G-to-A and C-to-U single nucleotide substitutions in the HCV genome, with an ensuing reduction of HCV RNA expression and HCV core antigen production. Ribavirin triphosphate (RTP) was dephosphorylated *in vitro* by recombinant ITPase to a similar extent as ITP, a naturally occurring substrate of ITPase, and reducing *ITPA* expression in Huh 7.5 cells by siRNA increased intracellular levels of RTP in addition to increasing HCV mutagenesis and reducing progeny virus production. Our results extend the understanding of the biological impact of reduced ITPase activity, demonstrate that RTP is a substrate of ITPase, and may point to personalized ribavirin dosage according to *ITPA* genotype in addition to novel antiviral strategies.

**IMPORTANCE** This study highlights the multiple modes of action of ribavirin, including depletion of intracellular GTP and increased hepatitis C virus mutagenesis. In cell culture, reduced ITP pyrophosphatase (ITPase) enzyme activity affected the intracellular concentrations of ribavirin triphosphate (RTP) and augmented the impact of ribavirin on the mutation rate and virus production. Additionally, our results imply that RTP, similar to ITP, a naturally occurring substrate of ITPase, is dephosphorylated *in vitro* by ITPase.

## Abstract (precis)

This study highlights the multiple modes of action of ribavirin, including depletion of intracellular GTP and increased hepatitis C virus mutagenesis. In cell culture, reduced ITP pyrophosphatase (ITPase) enzyme activity affected the intracellular concentrations of ribavirin triphosphate (RTP) and augmented the impact of ribavirin on the mutation rate and virus production. Additionally, our results imply that RTP, similar to ITP, a naturally occurring substrate of ITPase, is dephosphorylated *in vitro* by ITPase.

## INTRODUCTION

Two variants of the ITP pyrophosphatase (ITPase) gene (*ITPA*), a missense variant in exon 2 (*rs1127354*, P32T) and a splice-altering single nucleotide polymorphism (SNP) in intron 2 (*rs7270101*, IVS2), have been demonstrated in a genome-wide association study (GWAS) to protect against ribavirin-induced hemolytic anemia during therapy with peginterferon in combination with ribavirin for hepatitis C virus (HCV) genotype 1 infection (1). In patients with chronic HCV genotype 2 or 3 infection receiving peginterferon and ribavirin, *ITPA* variants encoding reduced ITPase activity were found in approximately one-third of patients and were associated with reduced relapse risk following antiviral therapy despite lower plasma ribavirin concentrations (2).

**FIG 1:**
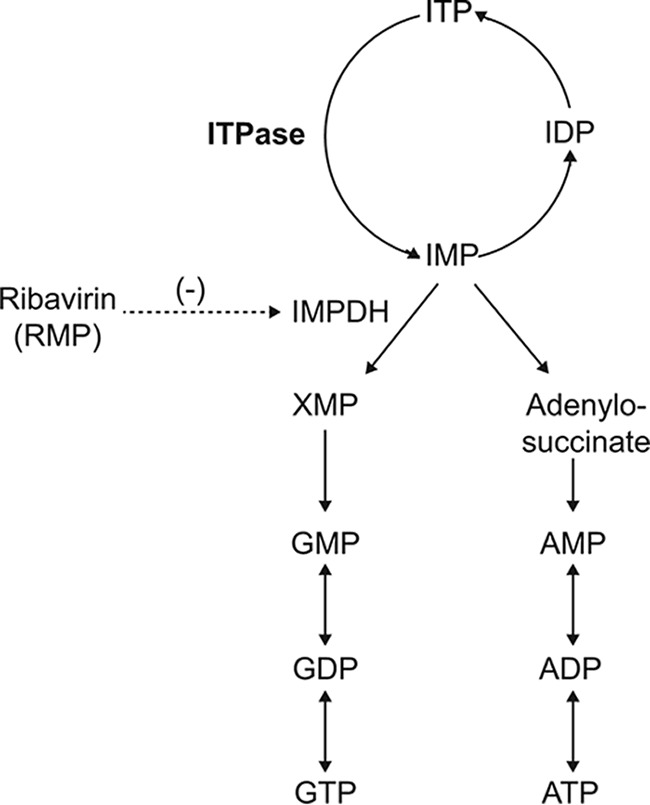
ITP to GTP and ATP and the nucleotide salvage pathway.

The evolutionarily conserved ITPase is a cytosolic enzyme that recycles purines by the pyrophosphohydrolysis of ITP to IMP and thus protects against integration of noncanonical nucleotides such as ITP and XTP, as well as their deoxy forms, into RNA or DNA (Fig. 1). These nucleotides may otherwise be incorrectly incorporated to produce genetic instability, anomalous proteins, and altered ATP-dependent signaling (3–6). ITPase deficiency has been associated with augmented toxicity in patients receiving purine analogues such as 6-mercaptopurine and azathioprine (7, 8). Studies of human cell lines have ascribed ITPase a role in protection against DNA damage (9), and in genetic knockout mouse models, the absence of ITPase generates growth retardation with cardiac myofiber disarray and death *in utero* or within 2 weeks of birth (10).

Ribavirin originally was synthesized as a guanosine analogue (11) and was soon noted to exert broad-spectrum *in vitro* activity against many RNA and DNA viruses (12). In addition to its utility in HCV therapy, treatment with ribavirin is of benefit in severe respiratory syncytial virus infections (13), viral hemorrhagic fevers such as Lassa and Crimean-Congo (14, 15), and hepatitis E virus infections (16). Unaided, ribavirin is insufficient to achieve clearance of HCV infection (17, 18) and has a modest effect on systemic levels of HCV RNA, with mean reductions of approximately 0.5 log_10_ IU/ml (19, 20). In the previously commonly used interferon-based therapy for chronic HCV infection, the addition of ribavirin markedly increased the likelihood of achieving sustained virological responses (SVR; defined as undetectable HCV RNA 24 weeks after the completion of treatment) by means of reduced relapse risk (21, 22). Presently, the use of ribavirin in HCV therapy is diminishing, but it continues to be recommended for some interferon-free, direct-acting antiviral (DAA) treatments (23), especially for HCV genotype 3 infection (24). Among HCV patients with decompensated cirrhosis receiving the recently approved pan-genotypic combination of sofosbuvir and velpatasvir in the ASTRAL-4 trial, the highest SVR rate was observed among patients additionally receiving ribavirin although the difference among rates was not statistically significant (25). Thus, in the setting of decompensated cirrhosis (Child-Pugh B or C), ribavirin likely will remain a component of HCV therapy as protease inhibitors should not be used in these patients (24).

Ribavirin has a large distribution volume, an elimination that is dependent on renal function, and a long half-life requiring in excess of 4 weeks to reach steady state (26–28). Ribavirin is subsequently activated by intracellular phosphorylation into mono-, di-, and triphosphates and upon phosphorylation becomes irreversibly entrapped in erythrocytes. Ribavirin therapy is hampered by adverse effects, most importantly dose-dependent hemolytic anemia, possibly secondary to oxidative stress caused by posterior depletion of ATP in erythrocytes (29, 30).

The detailed molecular mechanism underlying the action of ribavirin on HCV infection remains unclear, but several mechanisms of action have been proposed, including inhibition of IMP dehydrogenase (IMPDH) by ribavirin monophosphate (RMP) with ensuing GTP depletion (31), inhibition of capping of viral RNA (32), direct inhibition of the viral RNA polymerase (33, 34), and modulation of host immune responses (35). Additionally, ribavirin was shown to trigger viral mutagenesis leading to error catastrophe by means of incorporation of ribavirin triphosphate (RTP) (34, 36, 37) although this mechanism has been disputed by other investigators (38). *In vivo* (39, 40) and *in vitro* (41, 42) studies also imply that ribavirin downregulates the expression of interferon-stimulated genes (ISGs) in addition to reducing systemic concentrations of liver enzymes (19) and IP-10 (also known as CXCL10) (20).

The aim of the present study was to elucidate the mechanisms of action of the previously reported *in vivo* impact of reduced ITPase activity on the outcome of HCV therapy by means of assessing the *in vitro* effect of reduced enzymatic activity in the presence or absence of ribavirin in an HCV culture system.

## RESULTS

**FIG 2:**
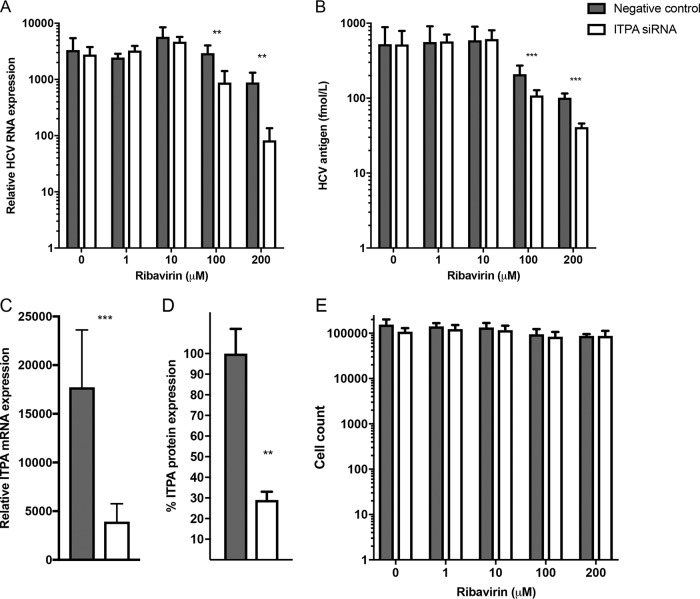
The effect of *ITPA* siRNA transfection and ribavirin treatment on HCV replication (A and B) and efficiency of *ITPA* siRNA transfection (C and D). (A) HCV RNA expression in HCV-infected Huh7.5 cells transfected with *ITPA* siRNA or a negative-control siRNA and ribavirin treatment as determined by real-time PCR and normalized to RPL4 expression. (B) HCV core antigen production in the supernatant of HCV-infected *ITPA* siRNA- or negative-control siRNA-transfected and ribavirin-treated Huh7.5 cells. (C) Efficiency of *ITPA* siRNA transfection on *ITPA* RNA expression in Huh7.5 cells transfected with *ITPA* siRNA or negative-control siRNA as determined by real-time PCR and normalized to RPL4 expression. (D) *ITPA* protein expression in Huh7.5 cells transfected with *ITPA* siRNA or negative-control siRNA and normalized to beta-actin protein levels as determined by immunoblotting. (E) Toxicity of ribavirin and siRNA treatment was determined by a proliferation assay of Huh7.5 cells treated with ribavirin and *ITPA* siRNA or a negative-control siRNA. Statistical significance was determined by a *t* test on logarithmic values (**, *P* < 0.01; ***, *P* < 0.001).

To mimic the previously reported *in vivo* impact of reduced ITPase activity, ranging from 60% to <5% of normal full activity (2), a small interfering RNA (siRNA) was utilized to decrease *ITPA* RNA expression *in vitro*. Huh7.5 cells were transfected with a pool consisting of four siRNAs directed against *ITPA* or a scrambled RNA (negative-control siRNA) for 48 h. Real-time PCR quantification of *ITPA* mRNA demonstrated that expression levels were significantly reduced to around 20% of the level observed in negative-control siRNA-transfected cells (Fig. 2C). Immunoblot detection of ITPase validated the effect of *ITPA* siRNA also on protein expression (Fig. 2D).

The HCV cell culture system was utilized to investigate effects of *ITPA* siRNA transfection and ribavirin on HCV replication. *ITPA* siRNA- or negative-control siRNA-transfected cells were incubated with graded concentrations of ribavirin (1 to 200 μM) and infected with the J6/JFH-1 HCV strain. Higher concentrations of ribavirin (100 to 200 μM) did not exert any effect on cell proliferation, and no difference was seen between *ITPA* siRNA-treated cells and those treated with a negative-control siRNA (Fig. 2E). Ribavirin exposure, as expected, decreased HCV RNA (normalized to expression of ribosomal protein L4, RPL4) and HCV core protein concentration levels in the infected cells transfected with a negative-control siRNA. Significant reduction in core antigen levels was seen in the 100 and 200 μM ribavirin-treated cells, and an HCV RNA reduction was seen in 200 μM ribavirin-treated cells compared to the level in untreated cells (*P* < 0.0005) (Fig. 2A and B). More interestingly, *ITPA* siRNA transfection in cells treated with 100 or 200 μM ribavirin significantly further reduced HCV RNA (*P* < 0.005) and HCV antigen (*P* < 0.0001) concentrations compared to levels with a negative-control siRNA (Fig. 2A and B). Decreased *ITPA* expression in HCV-infected cells thus promoted the ribavirin-induced reduction of HCV production, as measured by HCV RNA and HCV core antigen quantification.

**FIG 3:**
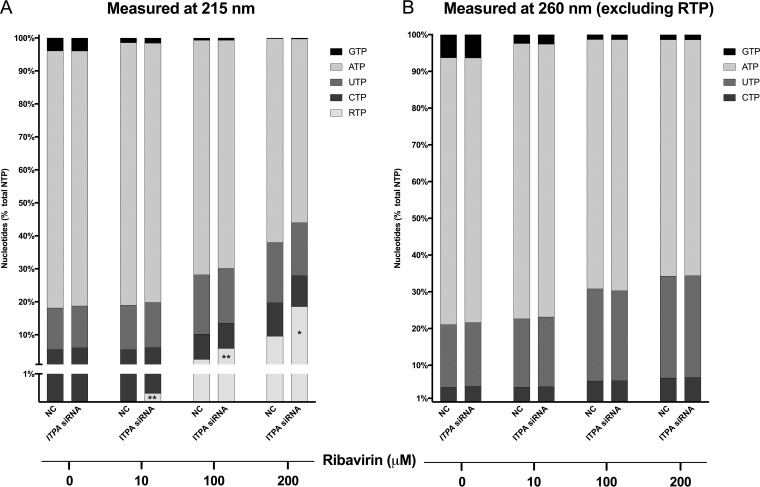
Intracellular CTP, UTP, ATP, GTP, and RTP amounts in *ITPA* siRNA- or negative-control (NC) siRNA-transfected and ribavirin-treated Huh7.5 cells detected by HPLC at 215 nm (A) and (excluding RTP) at 260 nm (B). Nucleoside triphosphate levels are quantified relative to the total amounts of nucleoside triphosphates. Statistical significance was determined by *t* test (*, *P* < 0.05; **, *P* < 0.01). Please note that RTP can be detected at 215 nm but not at 260 nm.

**FIG 4:**
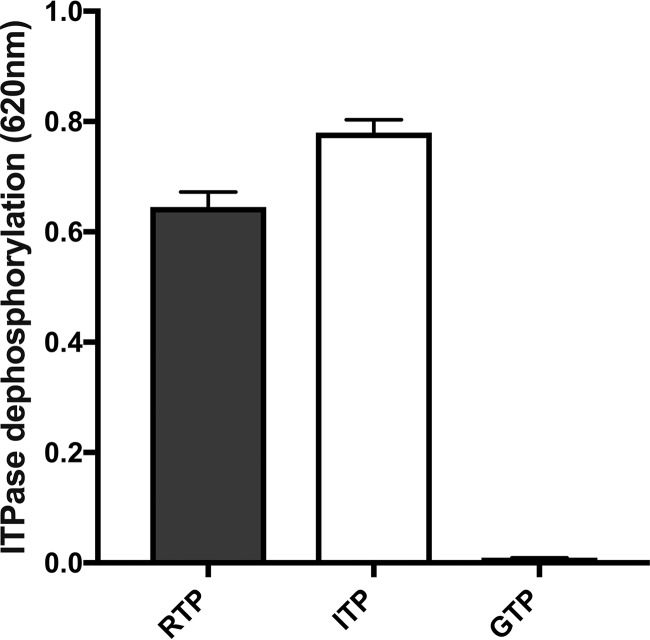
*In vitro* ITPase activity as measured by the dephosphorylation of RTP in comparison to that of ITP and GTP (positive and negative controls, respectively).

As both ITPase and IMPDH are involved in the nucleotide salvage pathway, the relative concentrations of all nucleoside triphosphates (NTPs) were measured in *ITPA* siRNA- or negative-control siRNA-transfected hepatocytes across a range of ribavirin concentrations. Quantification of cellular nucleotides at 260 nm showed that even a low ribavirin concentration (10 μM) profoundly decreased relative GTP levels (from 6.3% to 2.5% of the nucleotide pool; *P* = 0.0001). Higher concentrations of ribavirin (100 and 200 μM) resulted in a severe depletion of the GTP pool, combined with a modest decrease in relative levels of ATP and an increase in CTP and UTP. No significant differences were observed between *ITPA* siRNA- and negative-control siRNA-transfected cells with regard to relative GTP, ATP, CTP, or UTP concentrations (Fig. 3B). To also allow for the quantification of RTP, measurement of the samples was repeated at 215 nm (Fig. 3A). As expected, RTP concentrations increased with increasing ribavirin concentrations. However, *ITPA* siRNA transfection unexpectedly further increased RTP levels, resulting in approximately 19% of the total nucleotide pool consisting of RTP at 200 μM ribavirin in contrast to 10% for negative-control siRNA-transfected cells (*P* = 0.02) (Fig. 3A), suggesting that RTP is a substrate of ITPase, converting it into ribavirin monophosphate (RMP). To further explore this hypothesis, an *in vitro* phosphate detection assay using recombinant ITPase was used with RTP, ITP, and GTP as substrates. This assay confirmed the reactivity of ITPase for RTP, similar to that noted for ITP, a naturally occurring substrate of ITPase, whereas no reactivity was seen for GTP (Fig. 4). Together these data demonstrate that ITPase dephosphorylates RTP.

**FIG 5:**
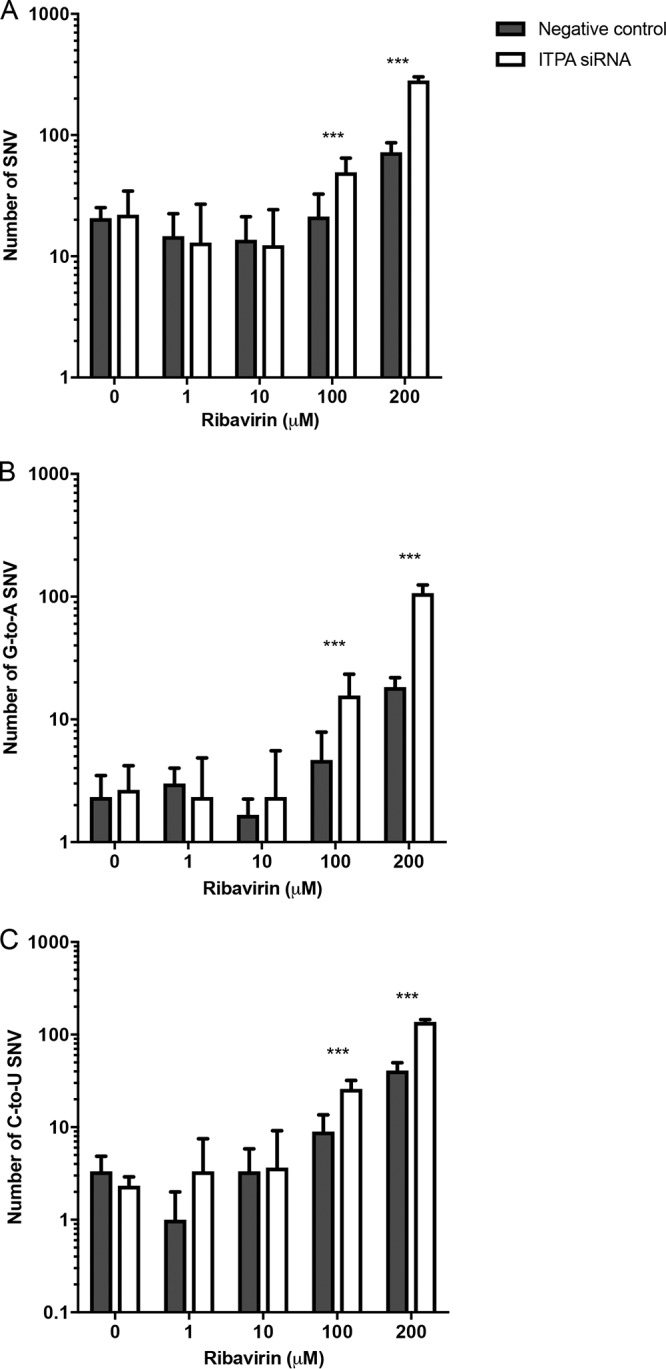
Single nucleotide variations (SNVs) (A) and G-to-A (B) and C-to-U (C) mutations across the HCV genome in HCV-infected Huh7.5 cells treated with ribavirin and transfected with *ITPA* siRNA or a negative-control siRNA. Statistical significance was determined by Poisson distribution (***, *P* < 0.001).

Next-generation sequencing (NGS) was used to determine mutations in the HCV RNA sequences, which were mainly C-to-U or G-to-A substitutions (Fig. 5A to C). Ribavirin at 200 μM dramatically increased HCV mutagenesis compared to levels with the negative-control siRNA-transfected cells (*P* < 0.001). Likewise, *ITPA* siRNA transfection resulted in increased mutagenesis already at 100 μM ribavirin and a prominent increase compared with the level for negative-control siRNA-transfected cells treated with 200 μM ribavirin, representing a significant difference for all C-to-U and G-to-A mutations (*P* < 0.005) (Fig. 5A). Interestingly, *ITPA* siRNA transfection led to a mutation pattern similar to that in negative-control siRNA-transfected cells treated with ribavirin but at a higher frequency. It should be noted, however, that while all the NGS analyses were sequenced at similar depths, the HCV RNA levels decreased when cells were treated with higher concentrations of ribavirin and transfected with *ITPA* siRNA (Fig. 2A). Indeed, NGS analyses of PCR amplimers may give rise to false perceptions of depth, especially if the original copy numbers are low.

**FIG 6:**
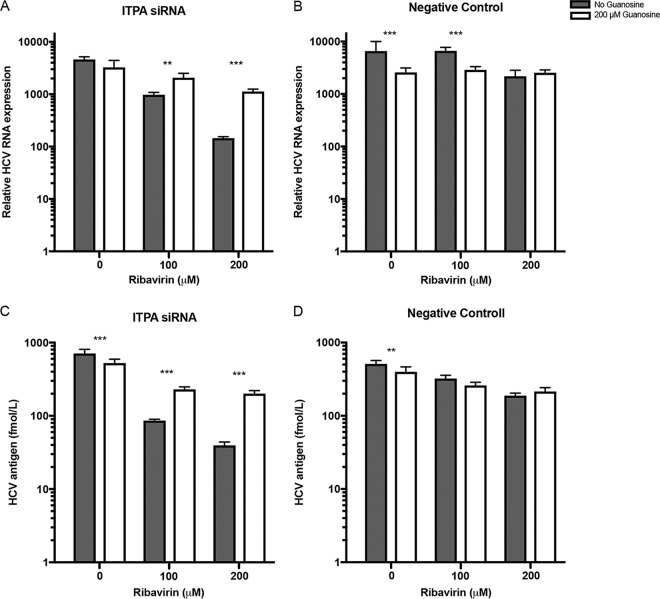
HCV RNA (A and B) and HCV core antigen (C and D) in Huh7.5 cells treated with ribavirin or ribavirin and guanosine and transfected with *ITPA* siRNA (A and C) or a negative-control siRNA (B and D). Statistical significance was determined by a *t* test on logarithmic values (**, *P* < 0.01; ***, *P* < 0.001).

To determine the impact of GTP depletion on the antiviral efficacy of ribavirin in *ITPA* siRNA- and negative-control siRNA-transfected cells, a guanosine salvage experiment was performed (Fig. 6). Guanosine supplementation significantly reduced the combined inhibitory effect of ribavirin and *ITPA* siRNA transfection as measured by HCV RNA expression (*P* < 0.01) and HCV core antigen quantification (*P* < 0.001). Of note, 200 μM guanosine supplementation significantly reduced HCV core antigen and HCV RNA when ribavirin was not added. Thus, GTP depletion may play a role in the combined antiviral activity of ribavirin and reduced ITPase activity.

## DISCUSSION

HCV-infected patients with reduced ITPase activity experience (i) reduced toxicity and (ii) improved efficacy of treatment with ribavirin, but the detailed mechanisms of ITPase and ribavirin interaction have remained unknown. This study was designed to clarify the impact of reduced ITPase activity on the actions of ribavirin in HCV-infected human liver cells. For this purpose, we introduced *ITPA* siRNA in HCV-infected Huh7.5 cells and achieved *ITPA* expression levels (20%) comparable to the level of ITPase activity reported in patients carrying genetic variants of *ITPA* encoding ITPase deficiency, i.e., activity of <5 to 60% of normal, wild-type ITPase activity. It was observed that the silencing of *ITPA* (i) promoted ribavirin-induced reduction in HCV RNA and HCV core antigen, (ii) potentiated the mutagenesis noted in the presence of higher concentrations of ribavirin, and (iii) increased the intracellular levels of RTP. Additionally, for the first time to our knowledge, ITPase was demonstrated to accept RTP as a substrate for dephosphorylation to RMP.

The effects of ribavirin on viral replication and mutagenesis were observed only at high doses of ribavirin (≥100 μM) that did not affect cell proliferation or viability, which is in line with a previous study showing a 50% inhibitory concentration (IC_50_) for ribavirin on J6/JFH-1 recombinant virus of 214 μM and an LC_50_ for ribavirin on Huh7.5 cells of 123 mM, with 90% live cells at 1 mM (43). Additionally, ≥10 μM ribavirin resulted in markedly reduced GTP levels, unrelated to *ITPA* siRNA, as well as an increase in RTP concentration when *ITPA* expression was reduced. Ribavirin at 10 μM is comparable to the steady-state concentrations achieved in patients during standard ribavirin weight-based dosing for HCV infection (20), whereas considerably higher ribavirin concentrations likely are achieved following dosing for Lassa virus infection (44). It should be noted that while patients generally achieve lower ribavirin plasma concentrations than those used in the *in vitro* HCV cell culture system in this study, patients are exposed to ribavirin for considerably longer times than the cells in the 72-h culture, which was the maximum time that the cells could be exposed without passage. Additionally, the Huh7.5 cell line is a hepatoma cell line and may differ from primary hepatocytes with regard to ribavirin metabolism.

Our finding that ITPase dephosphorylates RTP warrants further investigation. Interestingly, it has previously been reported that reduced ITPase activity is associated with lower plasma concentrations of unphosphorylated ribavirin in patients (2, 45), in spite of also being associated with improved outcomes and reduced toxicity. These clinical observations thus may be secondary to the association between reduced ITPase activity and increased intracellular RTP concentrations as the phosphorylation of ribavirin which occurs within cells leads to intracellular entrapment.

This study supports the previously suggested notion of multiple mechanisms of action of ribavirin (46); i.e., at low concentrations ribavirin markedly depleted intracellular GTP levels while inducing HCV mutagenesis at higher concentrations. The finding that low concentrations of ribavirin reduce GTP levels thus corroborates the previously reported competitive inhibition of IMP dehydrogenase (IMPDH) by RMP (47). Similarly, the observed increase in single nucleotide variations (SNVs) as ribavirin concentrations increased lends credence to the hypothesis that viral mutagenesis is caused by the incorporation of ribavirin triphosphate (RTP) (34, 36, 37) into the viral genome. Human polymerases, having proofreading capacity, are less likely to substitute RTP or noncanonical nucleotides for GTP. However, the profound reduction in intracellular GTP concentrations noted at relatively low ribavirin levels likely might have an additional impact on the levels of cellular gene transcription, as previously proposed (35, 39–42).

The majority of the mutations observed in this study using next-generation sequencing were G-to-A or C-to-U SNVs. This likely results from the incorrect substitution of RTP or ITP for GTP during the replication of viral RNA as intracellular GTP concentrations decline. Unlike GTP, RTP can form two hydrogen bonds with UTP or CTP with equal efficiencies (36), and ITP reportedly binds thermodynamically to CTP > ATP > UTP ≈ GTP (48). Either of these incorrect incorporations by the HCV RNA-dependent RNA polymerase, which lacks proofreading, would cause the observed mutation pattern. Although we did not document incorporation of RTP or ITP into the HCV genome, incorrect incorporation of RTP into the viral genome previously has been demonstrated for poliovirus and HCV (34, 36, 37). Additionally, recent *in vivo* evidence supports ribavirin-induced mutagenesis of the hepatitis E virus (HEV) genome (49). It should be noted that we did not detect quantifiable intracellular levels of ITP in this study despite repeated efforts, implying that the majority of the mutagenesis is driven by RTP rather than ITP incorporation, where increasing intracellular concentrations of RTP are observed in parallel with increasing numbers of SNVs.

The presence of intracellular ITP is a prerequisite for normal cellular function. It was long recognized that if only canonical pairing were allowed in nature, 64 species of tRNA would be required. However, empirically it was noted that most organisms have fewer than 45 species of tRNA, and thus some tRNA species must pair with more than one codon. To account for these observations, the wobble hypothesis postulated that the 5′ base on the anticodon in tRNA, which binds to the 3′ base on the mRNA, could have nonstandard base pairing (50). Similar to these *in vivo* observations in tRNA, replacement of canonical nucleotides by ITP often is utilized *in vitro* to induce wobbling, e.g., deoxy-ITP (dITP) may be incorporated into hybridization probes (51) or primers for PCR (52) at ambiguous codon positions to improve PCR efficiency. Additionally dITP may be incorporated *in vitro* to promote random mutagenesis (53) or directed evolution (54). In our study very few SNVs were noted in the absence of ribavirin. This may be an effect of *ITPA* siRNA not being able to completely silence expression of *ITPA* RNA or a result of the current experimental cell culture infections being standardized to 72 h. Greater reduction in ITPase activity or extending the duration of infection may have been required to document SNVs in the absence of ribavirin. Moreover, it is possible that relatively few substitutions in the viral genome, i.e., below the detection threshold in this study, could be sufficient to impact viral propagation, as previously noted for poliovirus, where low numbers of incorrect substitutions led to error catastrophe for the virus (34, 36, 37). The previous observation that the Cassava brown streak virus genome encodes an ITPase homologue (Maf/HAM1) (55) suggests that reduction of ITPase activity in infected cells in order to increase the mutagenesis of viral RNA may occur in some species and that some viruses might attempt to overcome this by increasing ITPase activity to maintain their genomic stability.

**FIG 7:**
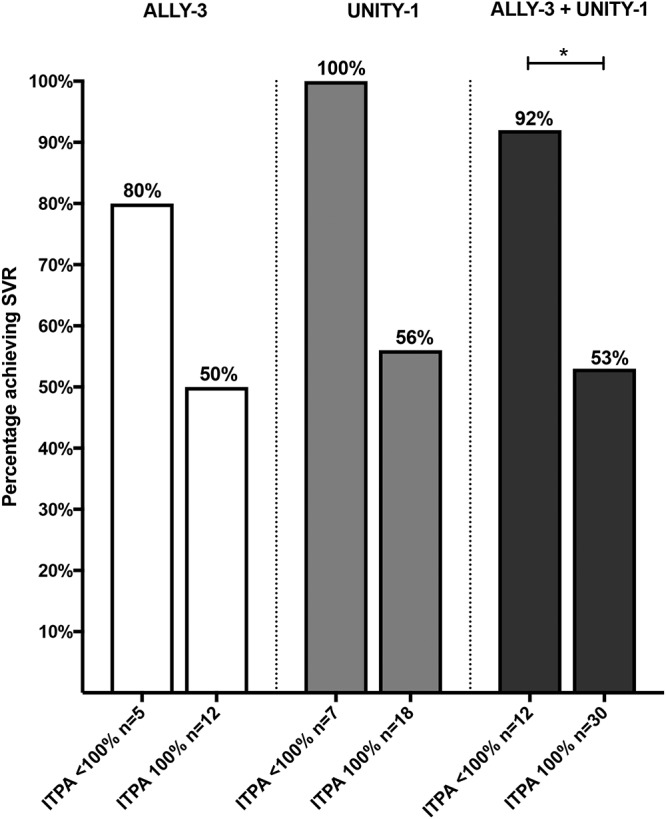
Percentage of patients with normal (100%) or reduced (<100%) ITPase activity having baseline, pretreatment NS5A resistance-associated substitutions (RASs; also known as resistance-associated variants, or RAVs) that achieved SVR following treatment with daclatasvir and sofosbuvir without ribavirin for 12 weeks in the ALLY-3 study (HCV genotype 3-infected patients with noncirrhosis or cirrhosis) and with daclatasvir, asunaprevir, and beclabuvir without ribavirin for 12 weeks in the UNITY-1 trial (HCV genotype 1-infected noncirrhotic patients). Statistical significance was determined using Fisher's exact test (*, *P* < 0.05).

Thus, stable or transient reduction of ITPase activity potentially may constitute a novel form of innate immunity in some organisms. This notion may be supported even in humans by observations among HCV-infected patients treated with ribavirin-free direct-acting antivirals (DAAs). Among 25 HCV genotype 1-infected, noncirrhotic patients treated with the interferon- and ribavirin-free combination of daclatasvir, asunaprevir, and beclabuvir for 12 weeks in the UNITY-1 trial (56) and having resistance-associated substitutions (RASs; also known as resistance-associated variants or RAVs) directed against the HCV protein NS5A at baseline prior to therapy, a nonsignificant trend toward improved outcome was observed in the seven patients having genetic variants encoding reduced ITPase activity (Fig. 7). A similar trend was observed among 17 HCV genotype 3-infected cirrhotic and noncirrhotic patients with baseline NS5A RASs/RAVs who were treated with daclatasvir and sofosbuvir without ribavirin for 12 weeks in the ALLY-3 study (57). Together, these studies demonstrate that in the presence of baseline NS5A RASs/RAVs, HCV-infected patients with reduced ITPase activity are significantly more likely to achieve SVR when treated with DAA therapy without ribavirin (*P* = 0.03 Fisher's exact test) (Fig. 7). Thus, even in the absence of ribavirin, reduced ITPase activity may beneficially impact the outcome of HCV infection. It should be noted, however, that the low number of subjects with RASs/RAVs limits the ability to make robust conclusions about the association of ITPase activity with the response in these subpopulations as ITPase activity was not associated with response on its own in these clinical trials.

The protection against ribavirin-induced anemia in patients with reduced predicted ITPase activity has been suggested to occur by means of avoidance of ATP reduction with ensuing diminution of oxidative stress and hemolysis (58). Interestingly, no major impact on ATP concentrations was detected in the presence or absence of ribavirin, and, likewise, ATP was not affected by the presence or absence of *ITPA* siRNA. This observation likely is secondary to differences in enzymatic activities between the hepatocyte-derived cells used in this study and erythrocytes. Also, in the present study guanosine supplementation partially reversed the effect of *ITPA* siRNA transfection on HCV propagation *in vitro*. This finding is coherent with previous reports on attempts to reverse the effects of ribavirin by guanosine supplementation where full reversion was not achieved (34, 36, 37).

The main clinical impact of the present study is further support for the notion that dosing of ribavirin likely may be individualized based on predicted ITPase activity rather than merely on weight and renal function, as is the current norm (59), although this hypothesis warrants confirmation in a prospective randomized trial. The findings from the NORDynamIC study, enrolling 382 HCV genotype 2- or 3-infected patients treated with standard 800-mg dosing of ribavirin in combination with interferon, demonstrated that patients carrying *ITPA* variants encoding reduced ITPase activity showed increased treatment efficacy mediated by reduced relapse risk and less anemia (2). Thus, development of a partial inhibitor of ITPase conceivably may improve the antiviral efficacy of ribavirin and attenuate its adverse effects. Such an inhibitor of ITPase could also be a potential antiviral agent in the absence of ribavirin.

In conclusion, this study demonstrated that the introduction of *ITPA* siRNA in an HCV cell culture system aiming at mimicking the reduced ITPase activity previously reported among one-third of humans (2) (i) reduced progeny virus production as measured by HCV core antigen and HCV RNA expression, (ii) increased ribavirin-induced mutagenesis, and (iii) increased the intracellular concentrations of ribavirin triphosphate. Our results demonstrate that RTP is a substrate of ITPase, extend the understanding of the biological impact of reduced ITPase activity, and may point to personalized ribavirin dosage according to *ITPA* genotype in addition to novel antiviral strategies based on modulation of ITPase activity.

## MATERIALS AND METHODS

### Virus and cells.

Huh7.5 cells (Apath, New York, NY) were cultured in cell culture medium consisting of Dulbecco's minimum essential medium (DMEM; Gibco, Carlsbad, CA) supplemented with 10% fetal calf serum (FCS), 1% nonessential amino acids (NEAA; Sigma, St. Louis, MO), and 1% penicillin-streptomycin. Huh7.5 cells are of wild-type *ITPA* genotype. An HCV J6/JFH-1 plasmid (kindly provided by Charles M. Rice, The Rockefeller University, New York) was used for production of infectious HCV.

### Production of HCV.

MEGAscript T7 RNA polymerase (Ambion, Carlsbad, CA) was used for *in vitro* transcription of a J6/JFH-1 plasmid according to the manufacturer's instructions. Briefly, 1 ng of plasmid was linearized with Xbal (Fermentas, Waltham, MA); the reaction was terminated, and the DNA was precipitated using 1/20 volume of 0.5 M EDTA, 1/10 volume of 5 M ammonium acetate, and 2 volumes of ethanol and incubated at −20°C for at least 15 min. The linearized plasmid was pelleted and resuspended in double-distilled H_2_O (ddH_2_O), and RNA was transcribed with T7 RNA polymerase at 37°C for 10 h, according to the manufacturer's protocol, with the addition of 1 μl of RiboLock RNase inhibitor (ThermoFisher Scientific, Carlsbad, CA). RNA was purified with an RNeasy RNA purification kit (Qiagen, Hilden, Germany) according to a standard protocol. The RNA was quantified using a NanoDrop 2000 instrument (Thermo Scientific, Carlsbad, CA). RNA quality was analyzed on a 1% agarose gel with SYBR green II RNA gel stain (ThermoFisher Scientific, Carlsbad, CA). Four million Huh7.5 cells were washed in buffered NaCl and resuspended in 400 μl of CytoMix (10 mM K_2_HPO_4_/KH_2_PO_4_ with 120 mM KCl, 0.15 mM CaCl_2_, 2 mM EGTA, 25 mM HEPES, 5 mM MgCl_2_, pH 7.6, and 2 mM ATP). Ten micrograms of RNA was added to the cells in a 0.4-cm cuvette and electroporated (960 μF, 260V) (Gene Pulser II; Bio-Rad, Hercules, CA). Twenty milliliters of cell culture medium was added to the cells, which were cultivated in six-well plates for 96 h. The cell culture medium was collected, and cell debris was removed by centrifugation at 350 × *g* for 7 min.

### HCV culture system.

During HCV infection, Huh7.5 cells were pretreated with ribavirin (Sigma, St. Louis, MO) at 2 h prior to infection. When cells were treated with guanosine (Sigma, St. Louis, MO), 200 μM guanosine was added simultaneously with ribavirin. Cells were infected with 15 pmol/liter HCV from cell culture medium, as quantified by HCV core antigen measurement, in 300 μl in 12-well plates. After 4 h, cells were washed three times with buffered NaCl and medium, or medium supplemented with ribavirin was added. Cells were analyzed at 72 h postinfection.

### siRNA treatment.

Huh7.5 cells were seeded at 1 × 10^5^ cells per well (12-well plate) in DMEM, 10% FCS, and 1% NEAA. Lipofectamine (ThermoFisher Scientific, Carlsbad, CA) and Opti-Mem (Gibco, Carlsbad, CA) were used for transfection of siRNA according to the manufacturer's instructions. The cells were transfected with four ITPA siRNAs (ITPA4, -5, -6, and -7) (Qiagen, Hilden, Germany) and a negative-control siRNA (scrambled RNA; Qiagen, Hilden, Germany) and incubated for 48 h.

### Antigen measurement.

Supernatant from each HCV-infected well was analyzed in duplicate with an Architect HCV Ag assay (Abbott, Chicago, IL) for automated detection of HCV core antigen (*n* = 12 for the experiments shown in Fig. 2B, *n* = 8 to 14 for the experiment shown in Fig. 6 C, and *n* = 8 to 14 for the experiment shown in Fig. 6D in each group).

### HCV RNA isolation.

Infected Huh7.5 cells were harvested by adding RLT lysis buffer (Qiagen, Hilden, Germany), and RNA was isolated using an RNeasy RNA isolation kit (Qiagen, Hilden Germany) according to the manufacturer's instructions. The purified RNA was treated with Turbo DNase (Ambion, Carlsbad, CA) at 37°C for 30 min for removal of cellular DNA.

### Real-time PCR.

The RNA (*n* = 6 in each group) was quantified with a NanoDrop 2000 instrument (ThermoFisher Scientific, Carlsbad, CA), and all samples were diluted to 20 ng/μl. Real-time PCR was performed using a Superscript III Platinum One-Step quantitative reverse transcription-PCR (qRT-PCR) kit with 6-carboxy-X-rhodamine (ROX) (Invitrogen, Carlsbad, CA) and with 40 ng of RNA, 500 nM primer, and 300 nM probe to detect ribosomal protein L4 (*RPL4*), *ITPA*, and HCV RNA. Primers and probes detecting HCV were used as previously published (60), and primers and probes detecting *RPL4* and *ITPA* were purchased from ThermoFisher Scientific. Expression was calculated using the Δ*C_T_* (where *C_T_* is threshold cycle) method, with *RPL4* used as a reference gene (61).

### Immunoblotting.

SDS-PAGE and immunoblotting were performed as previously described (62). Briefly, ITPA siRNA- and negative-control siRNA-transfected Huh7.5 cells were harvested in SDS sample buffer under reducing conditions and separated on a gradient polyacrylamide gel (Invitrogen, Carlsbad, CA). The proteins were transferred to polyvinylidene fluoride membranes (Millipore, Billerica, MA) and detected using rabbit antibodies against *ITPA* (1/2,000; Abcam, Cambridge, United Kingdom) with beta-actin (1/6,000; Abcam) as the control. Secondary anti-rabbit IgG peroxidase-conjugated antibodies (1/5,000; Abcam, Cambridge, United Kingdom) were used with SuperSignal West Dura Extended Duration Substrate (ThermoFisher Scientific, Carlsbad, CA). The detection of bands was viewed using a ChemiDoc MP System (Bio-Rad, Hercules, CA), and band intensities were calculated using ChemiDoc MP software, with ITPA protein expression normalized to beta-actin levels.

### Proliferation assay.

Huh7.5 cells of low confluence were cultured in medium supplemented with ribavirin and/or following *ITPA* siRNA transfection by trypsinization, and quantification of the number of cells present after treatment in comparison to the number of cells seeded at the start of treatment was performed.

### HPLC.

Huh7.5 cells were cultured in 10-cm dishes and transfected with *ITPA* siRNA or a negative-control siRNA (*n* = 4 in each group), as described above. Cells were harvested after 72 h and washed with cold-buffered NaCl. The cell material was collected using a cell scraper in 15% trichloroacetic acid (TCA) and 30 mM MgCl_2_ and snap-frozen in liquid nitrogen. Standard NTPs and intracellular ribavirin triphosphate (RTP) were extracted and measured as previously described (63). Briefly, NTPs were neutralized with two rounds of Freon-trioctylamine extraction, adjusted to pH 3.4, and analyzed on a LaChrom Elite high-performance liquid chromatography (HPLC) system (Hitachi, Tokyo, Japan) with a Partisphere SAX column (Hicrom, Berkshire, United Kingdom). To enable measurement of RTP, detection was carried out at 215 nm. The level of each nucleotide is expressed as a percentage of the total sample NTPs.

### Next-generation sequencing (NGS).

Isolated RNA (*n* = 4) was reverse transcribed using Superscript III (Invitrogen, Carlsbad, CA) with HCV-specific primers in *NS4A* (GCATTCCTCCATCTCATCAAAAGC) and *NS5B* (CTACCGAGCGGGGAGTAGGAAGAG). The cDNA was amplified in a nested PCR using Platinum HiFi Taq polymerase (Invitrogen, Carlsbad, CA) along with primer pairs for the 5′ (forward, ATGAGCACAAATCCTAAACCTCAAAGA, and reverse, GCATTCCTCCATCTCATCAAAAGC) and 3′ regions (forward, CCTCACACACATAGACGCCCACTT, and reverse, GTCCTGGGACGCCATGTGGAA) of the genome. The PCR was nested with forward primer TCAAAGAAAAACCAAAAGAAACACCAACCG along with reverse primer GAAACGCATCCAGTCGCCAGGCAA or GTCCTGGGACGCCATGTGGAA and GAAAAGTAGGAGTAGGCCGAAGAGTAATGA, respectively. The PCR product was visualized on a 1% agarose gel, and concentrations were measured with a NanoDrop 2000 instrument. The 5′ and 3′ PCR products were pooled, barcoded, and size selected to 200 bp using an Ion Xpress Plus fragment library kit (Thermo Scientific, Carlsbad, CA) together with an AB Library Builder System (ThermoFisher, Carlsbad, CA). The generated libraries were then amplified and purified according to protocol before quantification, and quality was assessed using a High Sensitivity D1000 screen tape kit (Agilent Technologies, Santa Clara, CA) together with an Agilent 2200 TapeStation system (Agilent Technologies, Santa Clara, CA). Six barcoded libraries were pooled before being amplified, conjugated to sphere particles, and loaded on an Ion 314 chip using Ion Chef (ThermoFisher, Carlsbad, CA). The samples were sequenced on an Ion PGM System (ThermoFisher, Carlsbad, CA). The results were analyzed with CLCbio (Qiagen, Hercules, CA), where each barcoded sample was quality checked. The quality trim threshold was set at 0.01 (PHRED score of >25), and sequences above 30 nucleotides in length were aligned to the J6/JFH-1 strain. All sequences had a read depth of at least 1,000×. The aligned sequences were analyzed using low-frequency variation. To validate reproducibility and to check for PCR errors, the first PCR and the nested PCR were sequenced for five samples, resulting in a comparable number of mutations, insertions, and deletions in the two PCRs run from the same sample (data not shown). The total number of detected mutations and the specific nucleotide substitutions were summarized for each sample.

### ITPase enzymatic assay.

Dephosphorylation of ITP, RTP, and GTP (*n* = 6) was analyzed using a PiColorLock Gold Phosphate detection system (Inova Biosciences, Cambridge, United Kingdom), according to the manufacturer's instructions. Nucleotides (0.1 mM) and 15 nM human ITPase (BioSite, San Diego, CA) in 50 mM Tris-HCl, pH 8, with 10 mM MgCl_2_ and 1 mM dithiothreitol (DTT) together with 0.5 U of inorganic pyrophosphatase (Roche Diagnostics, Risch, Switzerland), were incubated at 37°C for 10 min. Gold Mix was added to each well, followed by a stabilizer, and incubated in room temperature for 30 min. The plates were measured at 620 nm on a Multiskan FC (Thermo Scientific, Carlsbad, CA).

### Statistics.

One-way analysis of variance (ANOVA) with Dunnett's test was used to analyze differences in HCV-RNA, HCV antigen levels, and nucleotide concentrations between different treatments. A *t* test on logarithmic (when applicable) values was used to compare differences between results for ITPA siRNA-treated cells or guanosine-treated cells and those for controls. Differences in numbers of mutation were analyzed by Poisson distribution. Fisher's exact test was used to evaluate differences in cure rates of the ALLY-3 and UNITY-1 studies. Statistics were done in Prism (version 6.0c; GraphPad Software, La Jolla, CA) or SPSS (version 20.0.0; IBM Corp., Armonk, NY, USA) software. All reported *P* values are two sided, and *P* values of <0.05 were considered significant.
